# Group-Level Multivariate Analysis in EasyEEG Toolbox: Examining the Temporal Dynamics Using Topographic Responses

**DOI:** 10.3389/fnins.2018.00468

**Published:** 2018-07-17

**Authors:** Jinbiao Yang, Hao Zhu, Xing Tian

**Affiliations:** ^1^Neural and Cognitive Sciences, New York University Shanghai, Shanghai, China; ^2^Shanghai Key Laboratory of Brain Functional Genomics (Ministry of Education), School of Psychology and Cognitive Science, East China Normal University, Shanghai, China; ^3^NYU-ECNU, Institute of Brain and Cognitive Science, New York University Shanghai, Shanghai, China; ^4^Max Planck Institute for Psycholinguistics, Nijmegen, Netherlands; ^5^Centre for Language Studies Nijmegen, Radboud University, Nijmegen, Netherlands

**Keywords:** EEG, EEG/MEG, methodology, EEG signal processing, toolbox, topography, multivariate analysis, machine learning

## Abstract

Electroencephalography (EEG) provides high temporal resolution cognitive information from non-invasive recordings. However, one of the common practices–using a subset of sensors in ERP analysis is hard to provide a holistic and precise dynamic results. Selecting or grouping subsets of sensors may also be subject to selection bias, multiple comparison, and further complicated by individual differences in the group-level analysis. More importantly, changes in neural generators and variations in response magnitude from the same neural sources are difficult to separate, which limit the capacity of testing different aspects of cognitive hypotheses. We introduce EasyEEG, a toolbox that includes several multivariate analysis methods to directly test cognitive hypotheses based on topographic responses that include data from all sensors. These multivariate methods can investigate effects in the dimensions of response magnitude and topographic patterns separately using data in the sensor space, therefore enable assessing neural response dynamics. The concise workflow and the modular design provide user-friendly and programmer-friendly features. Users of all levels can benefit from the open-sourced, free EasyEEG to obtain a straightforward solution for efficient processing of EEG data and a complete pipeline from raw data to final results for publication.

## Introduction

Electroencephalography (EEG) is a suitable non-invasive measure for investigating the temporal dynamics of mental processing because of its high temporal resolution and cost-effectiveness. The event-related potential (ERP) is the most common way to reflect neural response dynamics in the temporal domain. However, ERP analyses are mostly based on responses in individual sensors or an average of a group of selected sensors. This “selecting sensors” analysis method is not optimal, because it faces various challenges (Tian and Huber, 2008; Tian et al., 2011). For example, only relying on data in a few sensors cannot easily differentiate between changes in the distribution of neural sources vs. changes in the magnitude of neural sources. Moreover, selecting sensors may introduce subjective bias during the selection processes, and sometimes data in different sensors may derive inconsistent or even contradicting results. Unless all possible sensor selections have been tested, readers will not know whether the reported effects are robust across sensors or sensor groups. Running statistical tests among multiple (groups of) sensors is subject to multiple comparisons, and hence increases the type I error (false positives) or type II error (false negatives that could be induced by correction methods). Furthermore, the ERP analysis heavily depends on identifying ERP components. However, data in a few sensors cannot fully represent the spatial and temporal features of components, which makes the estimation of components' response magnitude and latency hard and incomplete. Last, individual differences in spatial and temporal characteristics caused by anatomical and functional differences across subjects further complicate the analysis, which makes group-level analysis even more opaque. Therefore, most of the time, it is hard to get a precise and holistic view of temporal dynamics by using “selected sensors” in ERP analyses.

These problems may be solvable by using information from all available sensors. Two approaches can be taken. The first one is to localize neural sources by projecting all sensors information back to the source space (source localization). The advantage is that additional information about source spatial distribution can be estimated together with their temporal dynamics. Numerous source localization methods, such as dipole modeling, Loreta, Beamforming, and MNE (Grech et al., 2008), have been proposed and built in software packages such as BESA, EEGLab, Brainstorm, NutMEG, SPM, Fieldtrip, MNE-Python. However, source localization is an ill-posed problem–infinite solutions can be obtained from the mixture of recordings. Therefore, many assumptions have to be met and sophisticated procedures and careful manipulation have to be followed in order to obtain meaningful source localization results. Moreover, these localization methods work best with magnetoencephalography (MEG) that has better spatial resolution. EEG signals, on the other hand, are highly distorted by the skull. High-density EEG systems and realistic head models that are estimated by individual anatomical MRI scans are required to achieve acceptable results of EEG source localization. However, these high-cost systems and MRI scans may be not feasible for many researchers.

The second approach is to work with all “raw” data in the sensor space. Compared with methods with dependent variable from individual sensors or averages of selected sensors, this approach that relies on information from multiple sensors is called multivariate analysis. Basically, multivariate analysis in EEG uses the topographical patterns of sensors, and try to differentiate response patterns among conditions at each given time point. If differences, either in response magnitude, or topographic patterns, or latency were detected across a timespan, we can infer that different mental processes and their temporal dynamics mediate distinct conditions. This multivariate approach aims to directly test cognitive hypotheses by using data in all sensors (Tian and Huber, 2008; Tian et al., 2011) and by-passing source localization in case that the location information of cortical activities was not the primary research interest of the study. Note that performing the source localization by solving the inverse problem is the only way in EEG and MEG studies to directly address the questions regarding the location in the brain level. Scalp data and topographic patterns reflect the response dynamics at the sensor level and can be used as indicators of modulation by experimental manipulation.

In this paper, we introduce EasyEEG toolbox (https://github.com/ray306/EasyEEG), in which several multivariate analyses are included for processing EEG sensor data and testing cognitive hypotheses. To our knowledge, a few EEG analysis software packages (Delorme et al., 2011; Groppe et al., 2011; Pernet et al., 2011; Gramfort et al., 2013; Gerven et al., 2015) have already included several multivariate analysis methods for data in the sensor space. For example, LIMO EEG (Pernet et al., 2011) aims to test the effects at all sensors and all time points by a set of statistical tools such as ANOVA, ANCOVA and Hierarchical General Linear Model along with multiple comparisons corrections; Mass Univariate ERP Toolbox (Groppe et al., 2011) applies univariate tests (e.g., *t*-test) in each of all sensors over time points with multiple comparison correction; the Donders Machine Learning Toolbox (Gerven et al., 2015) supports the single-trial analysis on several machine learning methods built in, and MNE-Python (Gramfort et al., 2013) makes use of the a machine learn package named Scikit-Learn (Pedregosa et al., 2011) to see the decoding performance over temporal or spatial domain. Those toolboxes and the new toolbox EasyEEG shares the same goal which is to investigate the temporal neural dynamics using all data in all sensors. The uniqueness of EasyEEG toolbox is that the included multivariate methods are carried on the explicit measures that reflect the topographic patterns across all sensors. It offers a straightforward and intuitive approach to efficiently test cognitive hypotheses.

The designing principle of this toolbox is to be both user-friendly and programmer-friendly. So we separated the procedure of EEG data analysis into several steps, and made each step be an independent module with concise input/output interfaces. In each module, common important but tedious operations that involve complicated programming details have been encapsulated into several simple commands. Various multivariate group analysis methods have been built in with single lines of commands. Users simply need a descriptive dictionary to snip the data and one line of concatenated command to perform all analyses and visualize the results. After knowing only a few commands, all users, regardless of programming experience, could start their analysis within a few minutes. Moreover, the open-source nature of this toolbox enables and supports users to add more algorithms for the EEG data analysis. EasyEEG has encapsulated a lot of APIs for the programmers. The researchers who want to introduce a new analysis method should only pay the attention to the core logic of that method, but leave the trivial details, such as reshaping data and plotting, away from the programming. And even for the deep learning applications for EEG data, EasyEEG also provides a concise interface. In general, it offers a clear way to perform group level statistics tests to directly investigate cognitive hypotheses. We introduce how to use this package in the next section.

## Workflow and methods

The general analysis workflow in EasyEEG involves four stages:

1. Import the preprocessed data. EasyEEG currently (0.8.3) supports the epoches data generated from MNE and EEGLAB;
2. Define a dictionary (a Python syntax) to describe the analysis target (e.g., conditions, sensors, temporal durations, and/or any comparison between two groups), then extract the data by a function “extract()” with the definition as the parameter;
3. Apply one of four computation functions [e.g., “tanova()”] introduced in this paper. For algorithms that require long processing time, the computation process can be seen in a process bar showing used time and estimated rest time to finish; The computation function will yield a special data structure named *AnalyzedData*;
4. Visualize and output the results. *AnalyzedData* includes the name of analysis (in *analysis_name* attribute), the result of analysis (in *data, annotation* or *supplement* attribute), and the parameters for visualization (in *default_plot_params* attribute). Researchers can not only examine the *p*-values or other information, but also customize the visualization parameters for different figures.

You can see more detail in EasyEEG's online documentation (http://easyeeg.readthedocs.io/en/latest/).

We introduce a procedure that includes four multivariate methods for testing cognitive hypotheses using information in topographic patterns. An open dataset of face perception (Wakeman and Henson, 2015) is used to demonstrate this procedure and methods. The first two methods are to combine univariate approaches with topographic information to estimate the spatial extent of experimental effects (*distribution of significant sensors*) and the overall temporal dynamics of experimental effects (dynamics of global field power, *GFP*). These analyses can make the connection with common practice of ERP analysis. The next two methods are to implement multivariate analyses, introducing in this paper *topographic analysis of variance* (*TANOVA*) and *pattern classification that* take account of holistic topographic information to perform group-level statistics and investigate the dynamics of response patterns.

### Distribution of significant sensors

The spatial extent of experimental effects can be estimated by the number and distribution of sensors that are significantly different between conditions. This analysis is done by performing statistical tests, such as paired *t*-test, on response amplitude between two conditions in each sensor at all given time points or windows, and counting the number of the sensors that have significant results. In this way, we can quantify the spatial difference in terms of response amplitude between two topographies. By examining differences across timepoints, we can estimate the temporal dynamics of underlying neural processes that reflect in topographies.

### Dynamics of global field power (GFP)

Global field power (GFP) was introduced by Lehmann and Skrandies (Lehmann and Skrandies, 1980). It is calculated with the following equations (Equation 1):

(1) $$\begin{array}{l} GFP_{u}=\sqrt{\frac{1}{n}\cdot \sum_{i=1}^{n}u_{i}^{2}}\\ u_{i}=U_{i}-\bar{u}\\ \bar{u}=\frac{1}{n}\sum_{i=1}^{n}U_{i} \end{array}$$

where *n* is the number of sensors in the montage; *U*_*i*_ is the measured potential of the *i*th senosr (for a given condition at a given time point t); is the mean value of all *U*_*i*_; *u*_*i*_ is the average-referenced potential of the ith electrode.

Basically, GFP is a summary statistics of response magnitude from all sensors on a topography, which is in the form of variance of response magnitude and mathematically equals the root mean square (RMS) of all mean-referenced sensor values. GFP reflects the overall energy fluctuation of distributed electric potentials across all sensors at a specific time point. Therefore, it is a good way to summarize and visualize the temporal dynamics of the whole brain activities. Nevertheless, researchers need to be cautious that the essence of GFP is a non-linear transformation. Therefore, when researchers apply GFP to group-averaged ERP, the outcome is not the same as the average of individual GFPs. Variances between subjects have a major effect on group-averaged GFP.

The group-level statistical analysis of GFP can be addressed by many common approaches (time point by time point; area measures, peak measures etc.). We provide one of these approaches in the EasyEEG. For comparison between any two conditions, we take every subject's data from every temporal window with defined duration of interest from both conditions and apply paired *t*-test. Thus, we get the *p*-value that suggests the level of significance across all sensors in successive temporal windows.

### Topographic analysis of variance (TANOVA)

Topographies reflect underlying neural processes. Comparing pattern similarity between topographies in different conditions can reveal distinct mental processes and hence directly test cognitive hypotheses. TANOVA is a statistical analysis on a measure of similarity between topographies. This topographic similarity measure, called “angle measure” (Tian and Huber, 2008), where the topographic pattern similarity is quantified by a high-dimensional angle between two topographies. More specifically, the multivariate topographic patterns across all sensors are represented in high-dimensional vectors $\vec{A}$ and $\vec{B}$ for two conditions, where the number of dimensions is the number of sensors. The topographic similarity between the two conditions is quantified by the cosine value of the angle θ that can be obtained by the following equation (Equation 2).

(2) $$\begin{array}{l} cos\;\theta =\frac{\vec{A}\cdot \vec{B}}{\left|\vec{A}||\vec{B}\right|} \end{array}$$

The cosine value is an index of spatial similarity between two conditions, where the value of “1” represents identical patterns and value of “−1” represents exact opposite patterns. Moreover, because this index is normalized by response magnitude of both conditions, it has the advantage that it is unaffected by the magnitude of responses.

The statistical analysis of the “angle measure” is a non-parametric statistical test, termed topographic analysis of variance (TANOVA) (Murray et al., 2008; Brunet et al., 2011). The critical step in TANOVA is to generate a null distribution. In EasyEEG (0.8.4.1), we provided three different strategies to generate the null distribution of the angle measure cosine values.

Strategy 1:

1. Put all subjects' data into one pool regardless of experimental conditions.
2. Shuffle the pool and randomly re-assign a condition label for each trial (data permutation).
3. Calculate the group averaged ERPs for each new labeled condition.
4. Calculate the topographic similarity angle measure (cosine value of angle θ) between the new group-averaged ERPs.
5. Repeat the former steps (1–4) 1,000 times (suggested by Manly, 2006).

Strategy 2:

1. Perform data permutation within subject. That is, shuffle and re-label the trials for each subject.
2. Calculate the group averaged ERPs for each new labeled condition.
3. Calculate the topographic similarity angle measure (cosine value of angle θ) between the new group-averaged ERPs.
4. Repeat the former steps (1–3) 1000 times.

Strategy 3:

1. Calculate the ERPs for each condition and subject.
2. Perform data permutation at the within-subject level for ERPs. That is, re-label the ERPs for each subject.
3. Calculate the spatial topographic similarity angle measure (cosine value of angle θ) between for the new group- averaged ERPs.
4. Repeat the former steps (1–3) 1,000 times.

Strategy 1 is used by many researchers (Murray et al., 2008; Brunet et al., 2011; Lange et al., 2015). However, it loses subject's information by mixing all subjects' data into one pool. In contrast, Strategy 2 permutes the data at the within-subject level. Both Strategy 1, 2 may be time-consuming and computational demanding (about 8 h each strategy, reduced to 60 min when multithreading computation is applied. PC Configuration: CPU: Intel(R) Xeon(R) CPU E5-2667 v4 @ 3.20 GHz 32 cores; RAM: 256GB; System: Ubuntu16.04.1). Therefore, Strategy 3 has the advantage of reducing computing complexity and processing duration (can be done within 1–2 min). But Strategy 3 also has limitation that it loses trial information by averaging trials at the first step. Regardless of different procedures, we find out that the results from three strategies are similar and stable when the repetition times are beyond 1000 times (see details in the next section). Thus, we suggest that Strategy 3 can be used as a pilot test to have a quick check of results, and Strategy 2 for further validation.

After determining the null distribution, a comparison is made between the actual topographic similarity angle measure and the null distribution. The *p*-value is determined by finding the rank position of that actual cosine value in the generated null distribution. It reveals how significant the similarity between two topographic response patterns in different conditions are in a chosen time window.

### Pattern classification

Although TANOVA is good at detecting topographic variance at a given moment, it's insensitive to the fluctuation over time. We introduce a pattern classification method in EasyEEG to capture topographic dynamics. Moreover, pattern classification can collaboratively take advantage of all aspects of information in topographies, compared with GFP and TANOVA that only emphasize response magnitude and energy distribution, respectively.

This pattern classification method is in the framework of supervised machine learning. The collection of magnitudes of all sensors at a time point composes a sample, and the corresponding condition category is the label of the sample. After a classifier is trained by mapping the samples in a dataset to their labels, the classifier is used to infer the labels of samples in a new dataset for testing.

The pattern classification method aims for obtaining topographic differences among conditions at all timepoints to reveal the topographies changes over time. The general procedure work as follows:

1. Data in each condition in a specific time point or window are extracted to form a sample. Samples in the time points or windows of interest from two conditions form a dataset for each subject.
2. The pattern classification is done separately for each subject, so that we can obtain the classification results of all subjects at a given time point or window.
  2.1) Each dataset is divided into a training set and a test set. The samples in the training set are used to train the classifier, and then the samples in the test set are used to evaluate the trained classifier (get a classification score).
  2.2) Repeat step 2.1 for all time points and average the scores.
  2.3) Repeat step 2.1 and step 2.2 for each subject.
3. Compare the classification scores of all subjects with the chance level 0.5 for a two-alternative classification with the permutation test (Pitman, 1937). The *p*-value can be obtained to indicate whether topographies in two conditions are significantly different at a given time point or window.
4. Repeat the steps 2 and 3 at successive time points or windows, so that dynamics across time can be obtained.

Any supervised machine learning model can be used as a classifier. One should notice, however, that the classifier model determines the capacity of inferring the functional relationship between samples and their labels. The biggest issue for discovering the relationship is the number of available trials in the EEG data. In general, an EEG experiment generates fewer than hundreds of trials. If we attempt to infer a complex functional relationship from only a few hundreds of samples, the result can hardly generalize to other samples (the problem of “overfitting”). One solution is to keep the balance between the trial counts and the complexity of the functional relationship. For example, Logistics Regression (Cox, 1958) is a linear model, which can provide a simple functional relationship without much tuning of hyperparameters. We adopted the Logistics Regression algorithm as the default classifier model. Depending on different situations and needs, users can easily switch to other supervised machine learning algorithms such as Naive Bayes or Support Vector Machine in EasyEEG. Because sometimes the sample size in two labels might be unbalanced, we adopted Area Under Curve (AUC) as the classification score (King et al., 2013). And to make the classification score more robust, the algorithm will be applied to different partitions of the samples for several times (Cross Validation; Arlot and Celisse, 2010).

The simple classifier models can reduce overfitting, but the functional relationship they are able to catch may also be too simple to represent the real relationship. That is, some complicate topographic pattern differences won't be recognized by the model (the problem of “under-fitting”). The solution for under-fitting is to increase the complexity of classifier models which tends to cause overfitting. Therefore, we need to find a fine balance using appropriate regularization model (e.g., Krogh and Hertz, 1992; Prechelt, 1998; Hinton et al., 2012) or a special deep model that is designed for few samples (e.g., Kimura et al., 2018). Should one need to customize, all these extra optimizations can be easily added to the existing function by the programming interface provided in the toolbox.

## Examples and results

### Data for this tutorial

Data used for this tutorial are an open dataset of EEG responses to face stimuli (available at https://openfmri.org/dataset/ds000117/) (Wakeman and Henson, 2015). The face stimuli are made of 300 grayscale photographs (half from famous people and half from non-famous people) that are matched and cropped to show only the face. Additional 150 grayscale photographs of scrambled face that are generated by taking the 2D-Fourier transform of either famous or non-famous faces, permuting the phase information, and then inverse-transforming back into the image space. Subjects were required to make the judgment about how symmetric they regard each face stimulus by pressing a key, while EEG signals were recorded. The EEG data was acquired from by 16 healthy subjects at 1100 Hz sampling rate in a light magnetically shielded room using a 70 channel Easycap EEG cap (based on EC80 system: http://www.brainlatam.com/manufacturers/easycap/ec80–185). Full details about the experimental design and data acquisition can be found in Wakeman and Henson (2015)

### Processing pipeline

All raw data were first preprocessed by MNE-Python with a standard script (see Supplementary Code Snippet 1) and saved in the “.h5” format. Epochs were chosen from −200 ms pre-stimulus to 600 ms post-stimulus onset, and were baseline corrected based on the pre-stimulus period and band-pass filtered from 0.1 to 30 Hz. Epochs that contain artifacts were excluded based on a ±100μ*V* rejection criterion.

We demonstrate scripts for applying four analysis methods and their outcomes as follows (the entire script was running in a Jupyter notebook, see: https://github.com/ray306/EasyEEG/blob/master/tests/(Demo)%20Face%20perception.ipynb). The runtime environment for the following examples was based on EasyEEG 0.8.4.1, Python 3.6 64 bit, Ubuntu 16.04.1.

### Load data and define the analysis target

First, we define a dictionary that contains information for further analysis. The descriptive dictionary “target” is composed by two components: conditions and timepoints. To make the comparison between conditions, we add “&” between conditions as the operation symbol and use “X vs X” as the annotation. Because all analyses are based on all sensors, we don't need to define the channels. The duration of each epoch is 0–600 ms.

**Code Snippet 1:**
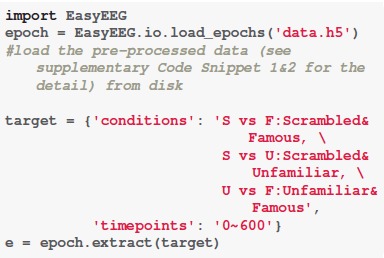
The data loading and analysis target definition.

The EasyEEG provides a simple and easy way to complete the loading and extraction process by calling the “load_epochs()” and the “extract()” functions. Data is extracted for further analysis by passing the descriptive dictionary “target” to the “extract()” function, and is saved in the variable “e.”

### Distribution of significant sensors

**Figure 1:**
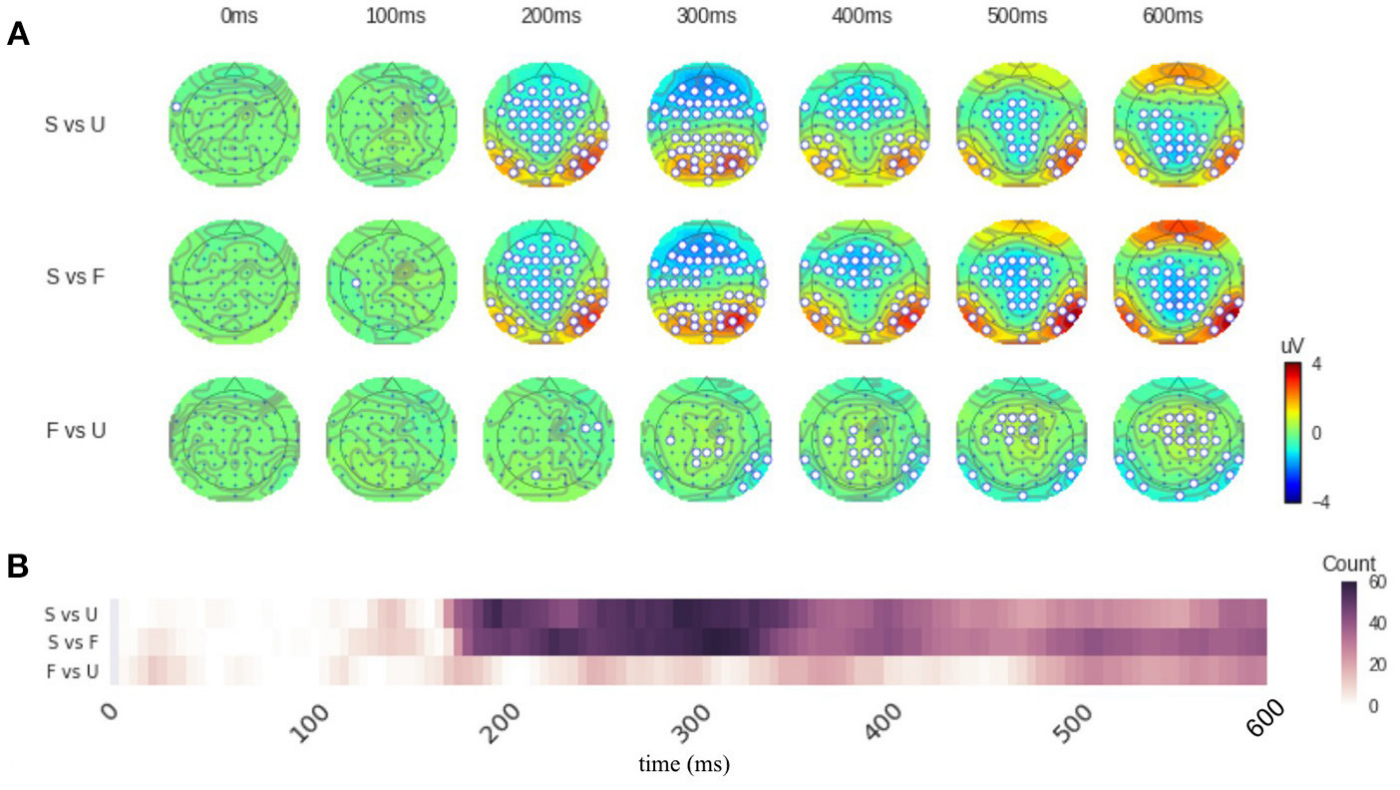
Results of *distribution of significant sensors* analysis. **(A)** Topographies of response differences between conditions across time. Each row contains topographies for a given comparison at different time points. Sensors that show significant response magnitude differences are circled in white. The color on the topography represents the response magnitude differences. The conditions in each comparison is listed on the left. S for scrambled condition, F for famous face condition, and U for unfamiliar face condition. **(B)** The number of significant sensors across time. The color scale represents the number of significant sensors. The conditions of comparison are listed at the left side of the figure. Labels are the same as in **(A)**. The comparison between face perception conditions (F and U) and scrambled (S) condition is significantly different in sensors above frontal, central, bilateral parietal-occipital areas, starting around 180 ms. The comparison between face perception conditions (F vs. U), however, only shows significant difference at the latencies of 300–400 ms and 500–600 ms. Refer to main text for detailed results.

By applying the function “*topography()*,” we can perform the distribution of significant sensors analysis. Specifically, we define successive time windows of every 100 ms. The distribution results are saved in the variable “result.” And by calling the function “*plot()*,” we can visualize the results (Figure 1). Sensors that show significant differences between two conditions are circled in white (Figure 1A). The function “*significant_channels_count()”* can be used to more clearly illustrate the temporal dynamics by the count of significant sensors. The results are saved in the variable “sig_ch_count” and depicted in Figure 1B that displays the number of significant sensors across time. The color scale represents the number of significant sensors.

**Code Snippet 2:**
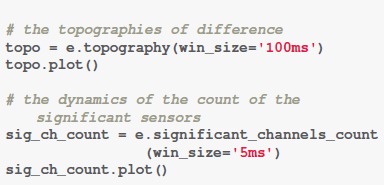
Apply the *Distribution of significant sensors* analysis.

Figure 1 shows that the comparison between conditions “Famous” (F) and “Scrambled” (S) as well as the comparison between conditions “Unfamiliar” (U) and “Scrambled” (S) are significantly different in sensors above frontal, central, bilateral parietal-occipital areas. These differences start around 200 ms (180 ms in sensor count results in Figure 1B). The comparison between conditions “Famous” (F) and “Unfamiliar” (U), however, only shows significant difference at the latencies of 300–400 ms and 500–600 ms. From 300 to 400 ms, only about 10 sensors above parietal and right-lateral occipital areas show significant differences. From 500 to 600 ms, around 25 sensors above middle frontal and bilateral occipital areas show significant differences. And these differences are weaker compared the comparisons between face and non-face conditions. See Supplementary Results 1, 2 for the summary of sensor magnitude, *p*-values, and the count of significant sensors. See supplementary ZIP file for the raw data.

### GFP

The function “*GFP()*” can be used to obtain the GFP. Computation of GFP can be done within a few seconds. We set the “*compare*” parameter to be “True” to enable statistical analysis between any two conditions. With the function “plot(),” the results of GFP can be visualized.

**Code Snippet 3:**
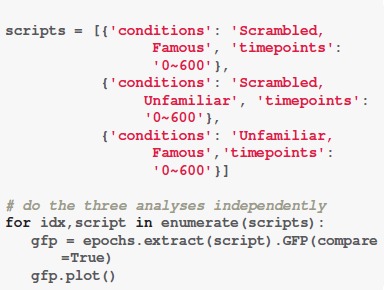
Apply the *GFP* analysis.

**Figure 2:**
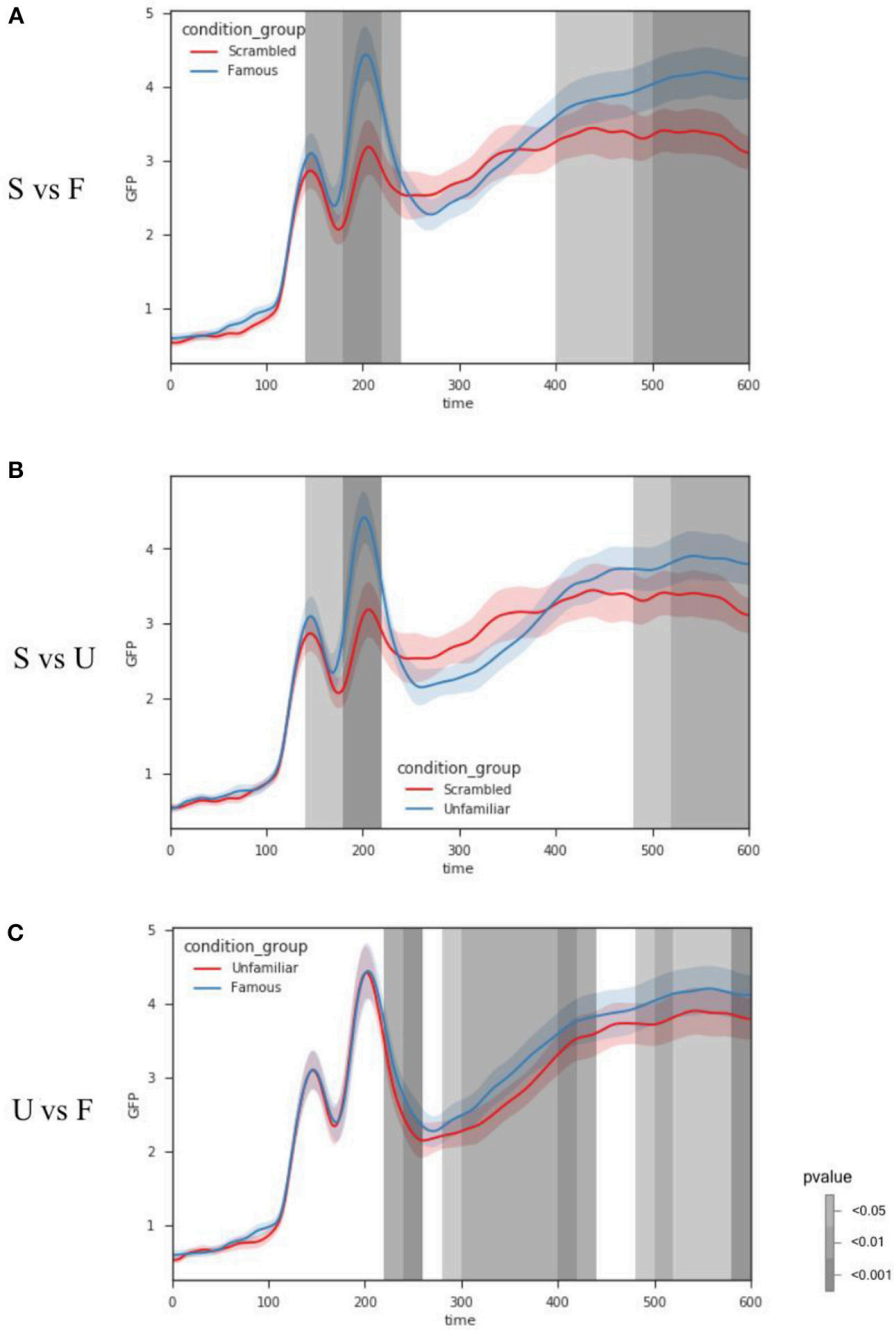
Results of *GFP* analysis. Each color line represents the GFP of each condition. Condition labels are the same as Figure 1. The shadow areas around each line depict the standard error of the mean. The grayscale vertical bar stands for the results of statistical analysis. Grayscale represents the significant levels, and location represents the latencies of significant effects. **(A,B)** The condition “Scrambled” (S) begins significantly different from the face perception conditions around 140 ms. Differences are also significant at some later latencies. **(C)** For comparison between two face perception conditions, significant differences are observed starting around 220 ms, later than those in comparisons between face and non-face conditions in **(A,B)**. Some later significant differences are also observed. Refer to main text for detailed results.

As shown in Figure 2, the condition “Scrambled” (S) begins significantly different from the condition “Famous” (F)” or Unfamiliar” (U)” around 140 ms. A small significant difference is found between conditions “Scrambled” (S) and “Unfamiliar” (U) at 500–600 ms, whereas the comparison between conditions “Scrambled” (S) and “Famous” (F) shows weak but significant difference at 400–600 ms. For comparison between conditions “Famous” (F) and “Unfamiliar” (U), significant differences are at 220–260 ms (most at 240 ms), 300–400 ms (most at 400 ms), and 500–600 ms (most at 600 ms). See Supplementary Result 3 for the summary of the GFP powers and the *p*-values over time. See supplementary ZIP file for the raw data.

### TANOVA

The function “*tanova()”* is for performing TANOVA analysis. Data was averaged in every 5 ms defined by the parameter “win_size.” The number of repetitions for creating the null distribution was set to 1,000 times as defined by the parameter “shuffle.” Different strategies of creating the null distribution can be defined by the parameter “strategy.” The computation time is about 60 times slower in Strategy 1 and Strategy 2 than that in Strategy 3 (about 1 min using our system). The output of “*tanova()”* function is the series of *p*-values. We corrected the *p*-values by accepting the consecutive significant data points which are longer than 20 ms (Lange et al., 2015) using a command “*correct(method* = '*cluster')*.” Users can also use the other solutions for multiple comparisons correction such as FDR Benjamini-Hochberg (Benjamini and Hochberg, 1995) by replacing the value of parameter “method.”

**Code Snippet 4:**
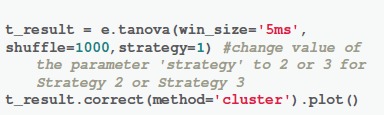
Apply the *TANOVA* analysis.

The results from Strategy 1 and Strategy 2 are highly similar. The topographic response patterns in condition “Scrambled” starts significantly different from those in the condition “Famous (F)/Unfamiliar (U)” after 170 ms (*p* < 0.01). For comparison between conditions “Famous” and “Unfamiliar,” most time after 470 ms are significantly different (*p* < 0.01) except from 530 to 560 ms. The results from Strategy 3 mostly agree with those from Strategy 1 and 2, with one noticeable exception at 180 ms for comparison between two face perception conditions. The results from all three comparisons show significant differences for a short time period around 180 ms (*p* < 0.01 for comparison “Scrambled vs. Unfamiliar” and comparison “Unfamiliar vs. Famous”; *p* < 0.05 for comparison “Scrambled vs. Famous”). See Supplementary Result 4 for the summary of the *p*-values of TANOVA over time. See supplementary ZIP file for the raw data.

### Pattern classification

The function “classification” is for performing pattern classification analysis. The default classifier is a logistic regression classifier. Data was averaged in every 5 ms defined by the parameter “win_size = ′5 ms”. The parameters “test_size = 0.3” and “fold = 25” indicate that the 30% of data were randomly selected as the test set and the rest are in the training set in each fold (data splitting iteration) and the number of folds is 25 in the cross validation.

**Code Snippet 5:**
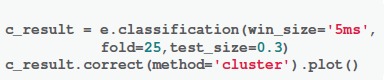
Apply the Pattern *classification* analysis.

Figure **4** depicts the pattern classification results as *p*-values across time. The condition “Scrambled” starts significantly different from those in condition “Famous (F) or Unfamiliar (U)” after 120 ms. The condition “Unfamiliar” and “Famous“ show sparse differences along time. More specifically, results show that at around 220 ms, 280 ms, 330 ms, 380 ms, 410–450 ms, and 510–600 ms, there are significant differences between these two conditions (*p* < 0.05). See Supplementary Result 5 for the summary of the scores of the *p*-values of Pattern classification over time. See supplementary ZIP file for the raw data.

The function “classification()” also allows researchers to use an external model such as a deep learning model (Chollet and Others, 2015; Abadi et al., 2016), see Supplementary Code Snippet 4 for an example.

## Discussion

EEG provides high temporal resolution information that reflects cognitive processes. However, common ERP methods using partial information in selected sensors are hard to obtain a precise and comprehensive temporal dynamics across the system. Whereas, source localization may estimate the distribution of neural generators and their dynamics. But sophisticated procedures, various assumptions, as well as high demand on data quality, facility and computational power may make localization methods not practical for some users. In the EasyEEG toolbox, we offer multivariate analyses that use EEG topographical patterns of sensors to obtain holistic system-level dynamic information without projecting back to the source space. Different types of analyses that take from distinct yet related perspectives help users infer different aspects of temporal dynamics by differentiating response patterns and magnitude across time. Main functions and other necessary steps have been packed in this toolbox, so that users can easily use. Moreover, the highly flexible, compatible and expandable design in programming are also ideal for advanced users. Our EasyEEG toolbox offers a practical, efficient and complete pipeline from raw data to publication for EEG research to directly test cognitive hypotheses.

This paper introduces four methods included in EasyEEG, which take information from all sensors of a topography to investigate neural dynamics. These methods yet target at different aspects of information and separately evaluate topographic patterns and response magnitude across time. The first method the distribution of significant sensors analysis can provide the spatial extent of effects by observing the spatial configuration and counting the number of sensors that have significant differences among conditions. The sample results show that greater spatial extent and more number of significant sensors in both face perception conditions, compared with scrambled condition, starting around 180 ms (Figure 1). These results indicate that the distribution of significant sensors can grossly identify the dynamics of neural processing in different conditions. The second method GFP analysis provides an indicator of overall energy variation among all sensors. The sample results show that the face perception conditions start to differ from scrambled condition around 140 ms, whereas response magnitudes differ between face perception conditions (famous vs unfamiliar) starting around 220 ms. These latency differences in response magnitude reveal that the general face perception occurs earlier, and specific face identification occurs later.

**Figure 3:**
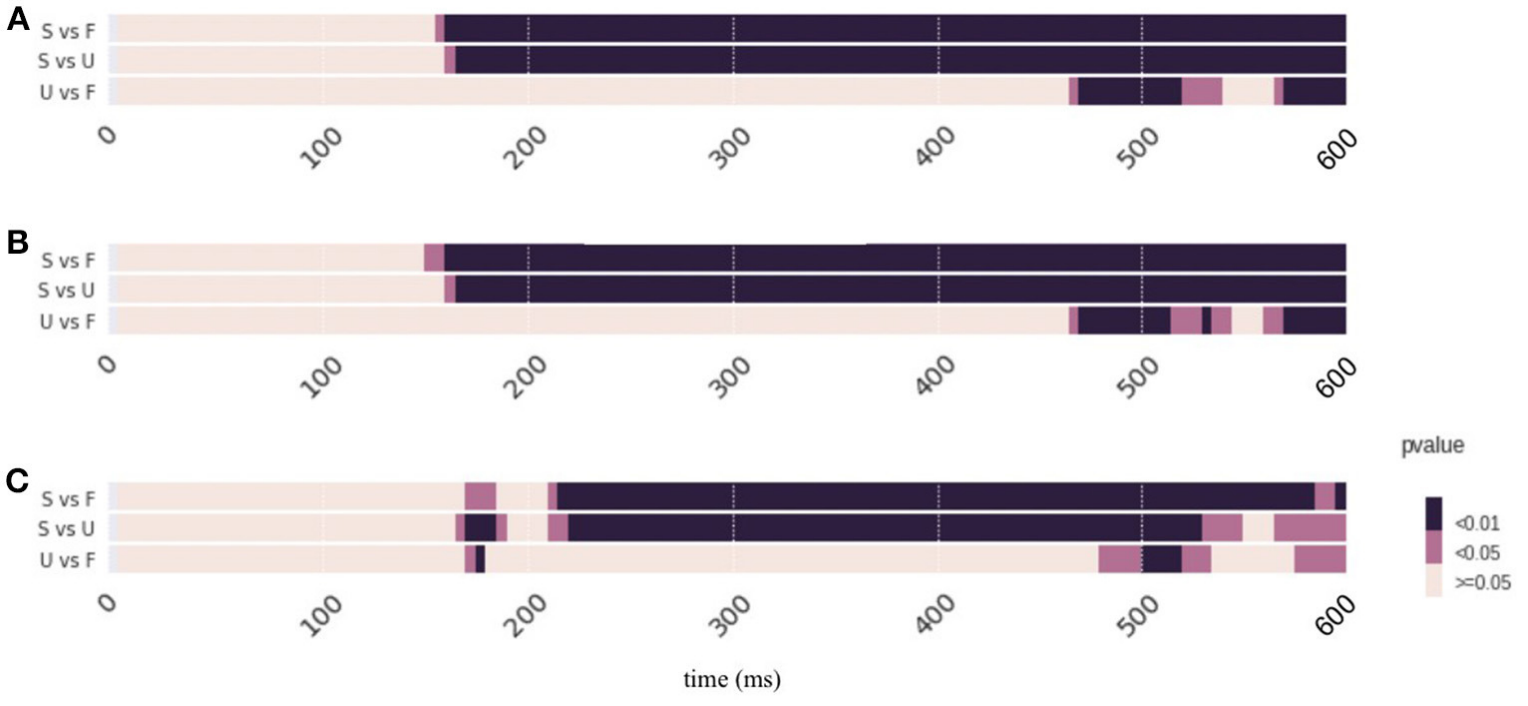
Results of *TANOVA* analysis. The results are represented as *p*-values across time. Color represents the significant levels, with darker color for smaller *p*-values. Conditions labels are the same as in Figure 1. **(A–C)** The results obtained by applying different strategies of computing null distribution in the non-parametric tests. These results are similar. The topographic response patterns in condition “Scrambled” starts significantly different from those in the face perception conditions after 170 ms and last till the end of epoch. For comparison between face perception conditions (F vs U), significant pattern differences are obtained after 470 ms. Results in Strategy 3 have an exception that all three comparisons show significant differences for a short time period around 180 ms. Refer to main text for detailed results.

The third method the TANOVA analysis provides a way to quantify and statistically test pattern similarity between topographies. The sample results show that the response topographic patterns in face perception conditions start to differ from those in scrambled condition around 170 ms (Figure 3). These results indicate that distinct processes for face perception emerge around 170 ms. Whereas, topographic responses in two face perception conditions remain the same until around 470 ms. These results indicate that similar sensor patterns mediate the perception of famous and unfamiliar faces during the early perceptual processes. The differences start around 470 ms could be because the effects of familiarity induce additional neural processes in famous condition compared with the processes for unfamiliar condition. The fourth method the pattern classification uses self-adaptive algorithms and takes advantage of all information regarding response magnitude and patterns of topographies to investigate neural dynamics. The sample results show that both face perception conditions show differences from the scrambled condition as early as around 120 ms. Differences between two face perception conditions are scattered across the timespan. These results indicate that the pattern classification method can reveal response magnitude and pattern differences as the classification results between two face conditions, as well as can provide additional information such as magnitude and pattern interaction, indicated by the detection of early differences between scrambled and face conditions.

These four methods are complementary to each other and can provide information at different levels to overcome limitation of individual methods. Users can use them collaboratively to obtain a comprehensive picture of their data. For example, the distribution of significant sensors was obtained by individually testing response magnitude differences in each sensor. This without correction is subject to multiple comparisons. We use this result to provide a general and direct visualization of data and dynamic results, similar to the common practice in fMRI research that uses “*p* < 0.05 uncorrected” for visualizing results.

The observed significant sensors distribution differences, as demonstrated in the face perception sample, can be caused either by response magnitude changes or the change of neural generators that is reflected in topographic patterns. We use the GFP and TANOVA to further test the magnitude and pattern differences among conditions, respectively. The GFP results show magnitude differences between two face perception conditions starting around 220 ms, whereas TANOVA results show pattern difference starting until 440 ms. These results from two methods collaboratively suggest that response magnitude in the same neural sources is firstly different between perceiving famous and unfamiliar faces, and later distinct neural generators are involved for processing familiarity. In the comparisons between face and scrambled conditions, both GFP and TANOVA analyses reveal differences start around 170 ms, suggesting both neural generators and their magnitude differ when processing faces or non-faces.

The pattern classification analysis gives the combination of magnitude and topographic differences, and can be used to verify and “double-check” the results in both GFP and TANOVA. In the sample results, the latencies of significant results in the classification agree with the combination of results in GFP and TANOVA in both comparisons between face and non-face conditions, as well as between face perception conditions. Moreover, the pattern classification can provide more information than GFP or TANOVA methods alone. This additional information is likely from the interaction between the response magnitude and patterns. For example, the early differences between face and scrambled conditions is only detected using pattern classification.

Based on the features of four methods and their complementary nature, we recommend the following procedure. User can follow all or partial of this procedure based on their research goals to obtain topographic and response magnitude dynamics.

1. Perform basic pre-processes such as noise reduction, baseline correction, filtering using other available toolboxes such as MNE Python.
2. Load the pre-processed data [*EasyEEG.io.load_epochs(“path”)*], define conditions and comparisons, and extract the data epochs of interests [*extract()*].
3. Obtain the distribution of significant sensors [*topography()*] for an direct and intuitive visualization [*plot()*] of effects.
4. Test the overall magnitude differences [*GFP().plot()*].
5. Test the topographic pattern differences [*tanova().plot()*].
6. Perform pattern classification [*classification().plot()*] to verify the results from (3) to (5).

By following the above 6 steps, users can visually inspect their data and effects, obtain the statistical results at the group-level regarding response magnitude and topographic patterns, and have a verification of obtained results from another perspective of pattern classification and machine learning. EasyEEG provides the realization of these steps and a complete pipeline from raw EEG data, to generating figures, to statistical testing for publication.

**Figure 4:**
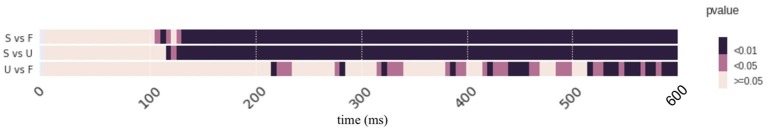
Results of *Pattern classification* analysis. Pattern classification results are represented as *p*-values across time. Color represents the significant levels. Condition labels are the same as in Figure 1. Both face perception conditions show differences (*p* < 0.01) from the scrambled condition as early as around 120 ms. Differences between two face perception conditions are scattered across the timespan. Refer to the main text for detailed results.

The results obtained by EasyEEG are consistent with those from other analysis approaches. A mass univariate General Linear Model (GLM) was applied on the same face perception dataset (Wakeman and Henson, 2015). Their results suggested that faces and scrambled conditions significantly differed from around 160 ms and last to the end of the epoch (600 ms), with differences in the sensors over fronto-central and lateral parieto-occipital areas, which are very consistent with our results (Figures 1, 2, 4). In the comparison between two face perception conditions, they found a single cluster over mid-frontal electrodes from 520 to 620 ms (Wakeman and Henson, 2015), which also agrees with our TANOVA results (Figure 3). These consistent results obtained by different approaches and toolboxes demonstrate the reliability of our methods and EasyEEG.

Besides the reliability, EasyEEG can obtain additional results and provide more insights. The most important one is separating response magnitude effects from topographic pattern changes. As in our results, GFP and TANOVA analyses reveal differences in response magnitude but not in topographic patterns between two face perception conditions, whereas both magnitude and patterns differ between face and scrambled conditions. These results highlight the advantage and capacity of EasyEEG on testing different aspects of hypotheses. Moreover, EasyEEG provides an unbiased omnibus measure using information of all sensors in topographies, which overcomes individual spatial and temporal differences and facilitates group-level analyses.

EasyEEG shares some attributes with other existing toolboxes of multivariate analyses, yet has distinct features. For instances, Mass Univariate ERP Toolbox applies the univariate test at each of all sensors, and reduces the multiple comparison pollution by different correction methods (Groppe et al., 2011); Whereas EasyEEG takes the topographical pattern of sensors directly with multivariate approaches, so that it can better avoid the multiple comparison problems than the univariate tests. LIMO EEG utilizes the hierarchical general linear model for multivariate data (Pernet et al., 2011), Donders Machine Learning Toolbox (Gerven et al., 2015) and MNE-Python offers an interface to Scikit-Learn for retrieving the classification score (Gramfort et al., 2013) a complete pipeline from the data loading and preprocessing to the statistical testing and results visualization.

EasyEEG offers great convenience and outstanding compatibility. The most common difficulty of using various software packages is how to get your own EEG data working in that toolbox. EasyEEG has a solution by reducing programming demands for customized algorithms. First, the complicated and tedious data extraction operations are replaced by calling built-in extraction function with descriptive dictionary. Researchers are only required to understand the structure of extracted EEG data. Second, EasyEEG makes extraction and combination of data in multiple sections/blocks automatic. In this way, users avoid the tedious and error-prone repetitive steps. Third, the proposed multivariate analysis methods have been implemented in simple command lines. Users can specify the intended analysis and parameters in one place and obtain the final results. Thus, researchers can focus more on their experiments and selection of core algorithms and methods, and obtain quick results to test their hypotheses.

EasyEEG also provides great flexibility and expandability for advanced users. Should researchers want to examine different aspects of data or to apply some other customized algorithms, they only need to modify a small portion of the current scripts to quickly create new computational or visualization algorithms based on a resilient data structure and a number of well-written application programming interfaces (APIs).

Besides the introduced multivariate analysis methods, we aim to include more analysis methods in EasyEEG to investigate neural dynamics, and increase the reliability of these methods. More specifically, we plan to integrate more machine learning models for EEG data analysis and pattern classification methods. Moreover, we aim to increase the efficiency and expandability of EasyEEG by designing more programming APIs for the developers.

There are several limitations of current version of our toolbox. First, methods included in our toolbox work best with the activation widely distributed among all sensors. However, if the effects are focused in several electrodes, the effect size could be reduced by the summary of topography, especially in the GFP analysis. Second, the multivariate methods rely on the topographies in the sensor space to infer the relation between neural sources of different conditions. The mapping between sources and topographies could be complicated. For example, two different neural sources, in theory, could generate the same pattern. If this situation occurred, our toolbox would derive incorrect results, although it is highly unlikely. Moreover, the topography-based analysis can find differences of neural sources between conditions. But it cannot further separate whether the differences are induced by the changing of source location or the orientation of the same source. All these limitations are induced by the cost-effectiveness tradeoff. While methods in our toolbox can offer direct and easy ways to test psychological and neuroscience, we sacrifice the ability to precisely testing aspects of underlying neural sources. Therefore, users should choose different methods based on their own questions and needs. Third, only four multivariate methods are built in the current version of toolbox. We are aiming to integrate more features in the future, such as deep learning techniques, to increase the power of our toolbox, meet broader requirement of users and provide solutions to wider ranges of questions.

In summary, EasyEEG provides simple, flexible and powerful methods that can be used to directly test cognitive hypotheses based on topographic responses. These multivariate methods can investigate effects in the dimensions of response magnitude and topographic patterns separately using data in the sensor space, therefore enable assessing neural response dynamics without sophisticated localization. Python based algorithms provide concise and extendable features of EasyEEG. Users of all levels can benefit from EasyEEG and obtain a straightforward solution to efficiently handle and process EEG data and a complete pipeline from raw data to publication.

## Author contributions

JY designed and programmed the toolbox. JY and HZ performed data analysis. HZ and XT advised on features and algorithms in the toolbox. JY, HZ, and XT wrote the paper. XT supervised this project.

### Conflict of interest statement

The authors declare that the research was conducted in the absence of any commercial or financial relationships that could be construed as a potential conflict of interest.

## References

[B1] Abadi M. Agarwal A. Barham P. Brevdo E. Chen Z. Citro C. (2016). TensorFlow: Large-Scale Machine Learning on Heterogeneous Distributed Systems. arXiv [cs.DC], 265–283. Available online at: http://arxiv.org/abs/1603.04467

[B2] Arlot S. Celisse A. (2010). A survey of cross-validation procedures for model selection. Stat. Surv. 4, 40–79. 10.1214/09-SS054

[B3] Benjamini Y. Hochberg Y. (1995). Controlling the false discovery rate: a practical and powerful approach to multiple testing. J. R. Stat. Soc. B Stat. Methodol. 57, 289–300.

[B4] Brunet D. Murray M. M. Michel C. M. (2011). Spatiotemporal analysis of multichannel EEG: CARTOOL. Comput. Intell. Neurosci. 2011:813870. 10.1155/2011/813870 21253358 PMC3022183

[B5] Chollet F. Others (2015). Keras. Available online at: https://keras.io/getting-started/faq/#how-should-i-cite-keras

[B6] Cox D. R. (1958). The Regression Analysis of Binary Sequences. J. R. Stat. Soc. B Stat. Methodol. 20, 215–242.

[B7] Delorme A. Mullen T. Kothe C. Akalin Acar Z. Bigdely-Shamlo N. Vankov A.. (2011). EEGLAB, SIFT, NFT, BCILAB, and ERICA: new tools for advanced EEG processing. Comput. Intell. Neurosci. 2011:130714. 10.1155/2011/130714 21687590 PMC3114412

[B8] Gerven M. Bahramisharif A. Farquhar J. Heskes T. (2015). Donders Machine Learning Toolbox, 2012. Available online at: https://github.com/distrep/DMLT. Last Accessed 1.

[B9] Gramfort A. Luessi M. Larson E. Engemann D. A. Strohmeier D. Brodbeck C.. (2013). MEG and EEG data analysis with MNE-Python. Front. Neurosci. 7:267. 10.3389/fnins.2013.00267 24431986 PMC3872725

[B10] Grech R. Cassar T. Muscat J. Camilleri K. P. Fabri S. G. Zervakis M.. (2008). Review on solving the inverse problem in EEG source analysis. J. Neuroeng. Rehabil. 5:25. 10.1186/1743-0003-5-25 18990257 PMC2605581

[B11] Groppe D. M. Urbach T. P. Kutas M. (2011). Mass univariate analysis of event-related brain potentials/fields I: a critical tutorial review. Psychophysiology 48, 1711–1725. 10.1111/j.1469-8986.2011.01273.x 21895683 PMC4060794

[B12] Hinton G. E. Srivastava N. Krizhevsky A. Sutskever I. Salakhutdinov R. R. (2012). Improving Neural Networks by Preventing Co-Adaptation of Feature Detectors. arXiv [cs.NE]. Available online at: http://arxiv.org/abs/1207.0580

[B13] Kimura A. Ghahramani Z. Takeuchi K. Iwata T. Ueda N. (2018). Imitation Networks: Few-Shot Learning of Neural Networks From Scratch. arXiv [stat.ML]. Available online at: http://arxiv.org/abs/1802.03039

[B14] King J. R. Faugeras F. Gramfort A. Schurger A. El Karoui I. Sitt J. D.. (2013). Single-trial decoding of auditory novelty responses facilitates the detection of residual consciousness. Neuroimage 83, 726–738. 10.1016/j.neuroimage.2013.07.013 23859924 PMC5635957

[B15] Krogh A. Hertz J. A. (1992). A simple weight decay can improve generalization, in Advances in Neural Information Processing Systems 4, eds Moody J. E. Hanson S. J. Lippmann R. P. (Burlington, MA: Morgan-Kaufmann), 950–957.

[B16] Lange V. M. Perret C. Laganaro M. (2015). Comparison of single-word and adjective-noun phrase production using event-related brain potentials. Cortex 67, 15–29. 10.1016/j.cortex.2015.02.017 25863469

[B17] Lehmann D. Skrandies W. (1980). Reference-free identification of components of checkerboard-evoked multichannel potential fields. Electroencephalogr. Clin. Neurophysiol. 48, 609–621. 10.1016/0013-4694(80)90419-8 6155251

[B18] Manly B. F. J. (2006). Randomization, Bootstrap and Monte Carlo Methods in Biology, 3rd Edn. New York, NY: Chapman and Hall/CRC.

[B19] Murray M. M. Brunet D. Michel C. M. (2008). Topographic ERP analyses: a step-by-step tutorial review. Brain Topogr. 20, 249–264. 10.1007/s10548-008-0054-5 18347966

[B20] Pedregosa F. Varoquaux G. Gramfort A. Michel V. Thirion B. Grisel O. (2011). Scikit-learn: machine learning in python. J. Mach. Learn. Res. 12, 2825–2830.

[B21] Pernet C. R. Chauveau N. Gaspar C. Rousselet G. A. (2011). LIMO EEG: a toolbox for hierarchical LInear MOdeling of ElectroEncephaloGraphic data. Comput. Intell. Neurosci. 2011:831409. 10.1155/2011/831409 21403915 PMC3049326

[B22] Pitman E. J. G. (1937). Significance tests which may be applied to samples from any populations. Suppl. J. R. Stat. Soc. 4, 119–130. 10.2307/2984124

[B23] Prechelt L. (1998). Early stopping-but when?, in Neural Networks: Tricks of the Trade, eds Orr G. B. Müller K.-R. (Berlin; Heidelberg: Springer Berlin Heidelberg), 55–69.

[B24] Tian X. Huber D. E. (2008). Measures of spatial similarity and response magnitude in MEG and scalp EEG. Brain Topogr. 20, 131–141. 10.1007/s10548-007-0040-3 18080180

[B25] Tian X. Poeppel D. Huber D. E. (2011). TopoToolbox: using sensor topography to calculate psychologically meaningful measures from event-related EEG/MEG. Comput. Intell. Neurosci. 2011:674605. 10.1155/2011/674605 21577268 PMC3090718

[B26] Wakeman D. G. Henson R. N. (2015). A multi-subject, multi-modal human neuroimaging dataset. Sci. Data 2:150001. 10.1038/sdata.2015.1 25977808 PMC4412149

