# Supplementary material for: Group-Level Multivariate Analysis in EasyEEG Toolbox: Examining the Temporal Dynamics Using Topographic Responses
Source: Front Neurosci. 2018 Jul 17;12:468. doi: 10.3389/fnins.2018.00468 (PMC6057229; doi:10.3389/fnins.2018.00468)
Supplement: Supplementary file 1 [file Table_1.pdf]

Supplymentary materials

Supplementary Code Snippet 1. Pre-processing script for MNE-Python.

```
import mne
from mne import find_events, Epochs, pick_types, read_evokeds
import pandas as pd
import numpy as np

ch_name_dic ={'EEG001': 'PO9', 'EEG002': 'Fpz', 'EEG003': 'PO10',
'EEG004': 'AF7',
              'EEG005': 'AF3', 'EEG006': 'AFz', 'EEG007': 'AF4', 'EEG008':
'AF8', 'EEG009': 'F7',
              'EEG010': 'F5', 'EEG011': 'F3', 'EEG012': 'F1', 'EEG013':
'Fz', 'EEG014': 'F2',
              'EEG015': 'F4', 'EEG016': 'F6', 'EEG017': 'F8', 'EEG018':
'FT9', 'EEG019': 'FT7',
              'EEG020': 'FC5', 'EEG021': 'FC3', 'EEG022': 'FC1', 'EEG023':
'FCz', 'EEG024': 'FC2',
              'EEG025': 'FC4', 'EEG026': 'FC6', 'EEG027': 'FT8', 'EEG028':
'FT10', 'EEG029': 'T9',
              'EEG030': 'T7', 'EEG031': 'C5', 'EEG032': 'C3', 'EEG033':
'C1', 'EEG034': 'Cz',
              'EEG035': 'C2', 'EEG036': 'C4', 'EEG037': 'C6', 'EEG038':
'T8', 'EEG039': 'T10',
              'EEG040': 'TP9', 'EEG041': 'TP7', 'EEG042': 'CP5', 'EEG043':
'CP3', 'EEG044': 'CP1',
              'EEG045': 'CPz', 'EEG046': 'CP2', 'EEG047': 'CP4', 'EEG048':
'CP6', 'EEG049': 'TP8',
              'EEG050': 'TP10', 'EEG051': 'P9', 'EEG052': 'P7', 'EEG053':
'P5', 'EEG054': 'P3',
              'EEG055': 'P1', 'EEG056': 'Pz', 'EEG057': 'P2', 'EEG058':
'P4', 'EEG059': 'P6',
              'EEG060': 'P8', 'EEG065': 'P10', 'EEG066': 'PO7', 'EEG067':
'PO3', 'EEG068': 'POz',
              'EEG069': 'PO4', 'EEG070': 'PO8', 'EEG071': 'O1', 'EEG072':
'Oz', 'EEG073': 'O2', 'EEG074': 'Iz'}

event_id = {'Famous':1,'Unfamiliar':2,'Scrambled':3}

for subID in range(1,17):
    path = f'/Users/ds000117_R1.0.0/sub-{subID:03}/ses-meg/meg'
```

```
    for runID in range(1,7):
        raw = mne.io.read_raw_fif(f'{path}/sub-{subID:03}_ses-meg_task-
facerecognition_run-{runID:02}_meg.fif',preload=True)

raw.set_channel_types({'EEG061':'eog','EEG062':'eog','EEG063':'ecg','EEG06
4':'misc'})
        # raw.plot(block=True)
        events =
mne.find_events(raw,stim_channel='STI101',shortest_event=1)
        events = mne.merge_events(events,[5,6,7],1)
        events = mne.merge_events(events,[13,14,15],2)
        events = mne.merge_events(events,[17,18,19],3)

        picks =
mne.pick_types(raw.info,meg=False,eeg=True,stim=False,eog=False,ecg=False,
misc=False)
        raw.filter(0.1,30,n_jobs=2,fir_design='firwin')

        epochs = mne.Epochs(raw, events, event_id, tmin=-0.2, tmax=0.6,
picks=picks,
                            baseline=(-0.2, 0), reject=None, preload=True)
        # epoch_raw.drop_channels(['EEG061','EEG062','EEG063','EEG064'])
        epochs.resample(1000.00)
        epochs.rename_channels(ch_name_dic)
        # epochs.plot(block=True)
        # epochs.info['bads'] = ['EEG061','EEG062','EEG063','EEG064']
        # epochs.interpolate_bads(reset_bads=True, mode='accurate',
verbose=None)
        epochs.set_eeg_reference(ref_channels='average', projection=True)
        epochs.apply_proj()

        eeg_reject = dict(eeg=200e-6)
        epochs.drop_bad(reject=eeg_reject, flat=None, verbose=None)

epochs.save(f'/Users/preprocessed/sub-{subID}-run-{runID}-eeg-epo.fif')
```

Supplementary Code Snippet 2. Load multiple '.fif' epoch files and save as one '.h5' file.

```
import easyEEG
path = '/Users/preprocessed/'
fif_list = [f'{path}sub-{subID}-run-{runID}-eeg-epo.fif'
              for subID in range(1,17) for runID in range(1,7)]
```

```
epochs = easyEEG.io.load_mne_fif(fif_list)
epochs.save('DATAS/EasyEEG_paper/data.h5')
```

Supplementary Code Snippet 3. Detailed code for "*topography()*". We should specify the "'channels':'each'" in the target definition.

```
scripts = [{'conditions': 'Scrambled,Famous',
                'timepoints': '0~600'},
           {'conditions': 'Scrambled,Unfamiliar',
                'timepoints': '0~600'},
           {'conditions': 'Unfamiliar,Famous',
                'timepoints': '0~600'}]

for idx,script in enumerate(scripts):
    e = epochs.extract(script)
    GFP = e.GFP(compare=True)
    GFP.default_plot_params['style']='ticks' # remove the backgroud
color for paper publication
    GFP.save(f'DATAS/EasyEEG_paper/gfp{idx}')
    GFP.plot()
```

Supplementary Code Snippet 4. An example for using an external classifier (Convolutional neural network). A simple classifier model may fail to fit the real relationship between data points and their condition labels, and so fail to recognize some complicate differences between conditions. We provide an example of how to build an complex classifier, Convolutional neural network (CNN), by Tensorflow (Abadi et al., 2016) and Keras (Chollet and Others, 2015). CNN is a type of deep learning model. It accepts feature matrices (topographical images) rather than the feature vectors (amplitudes), so we define the feature transformation in function "reshape_X" and pass the function as the value of parameter "reshape_X_method". Then we define the model structure and the measurement of model performance in function "run_model" and pass the function as the value of parameter "run_model". The model consists of two convolutional layers with ReLU-activation and max-pooling, a fully connected layer with ReLU-activation, and a fully connected layer with softmax-activation. The convolution layer can learn the interactions among sensors and robust to the EEG signal noise. Ideally the model should work better than Logistic Regression. However, we do not adjust parameters in this model for EEG data or apply few-shot technologies (learning from a small size of samples). Therefore, the results could be sub-optimal.

```
from EasyEEG.graph.figure_unit import get_topograph

import tensorflow as tf
from tensorflow.python.keras.models import Sequential
from tensorflow.python.keras.utils import to_categorical
from tensorflow.python.keras.layers import Reshape, MaxPooling2D,
Conv2D, Dense, Flatten
```

```
from tensorflow.python.keras.optimizers import Adam
from sklearn.metrics import roc_auc_score

# transform the feature vector (amplitudes) to a feature matrix
(topography)
def reshape_X(X, extra_params):
    N = 20
    locs = epochs.info['xy_locs']
    channels = X[0].columns.get_level_values('channel')

    X = np.array([get_topograph(i, locs, channels, N) for i in X])
    return X

# define a CNN model
def run_model(X, Y, train_index, test_index, extra_params):
    # model structure (https://github.com/Hvass-Labs/TensorFlow-
Tutorials/blob/master/03C_Keras_API.ipynb)
    img_size = 20

    model = Sequential()

    model.add(Reshape((img_size, img_size, 1), input_shape=(img_size,
img_size)))

    model.add(Conv2D(kernel_size=5, strides=1, filters=16,
padding='same', activation='relu'))
    model.add(MaxPooling2D(pool_size=2, strides=2))

    model.add(Conv2D(kernel_size=5, strides=1, filters=36,
padding='same', activation='relu'))
    model.add(MaxPooling2D(pool_size=2, strides=2))

    model.add(Flatten()) # Flatten the 4-rank output of the
convolutional layers to 2-rank that can be input to a fully connected
layer.
    model.add(Dense(128, activation='relu'))
    model.add(Dense(2, activation='softmax')) # Last fully connected
layer with softmax-activation, for the classification

    model.compile(loss='categorical_crossentropy', optimizer=Adam(lr=1e-
2))
```

```
    # model trainning
    model.fit(x=X[train_index], y=to_categorical(Y)[train_index],
batch_size=30, epochs=1, verbose=0)

    # model inference
    prob_train = model.predict_proba(X[train_index])[:,1]
    prob_test = model.predict_proba(X[test_index])[:,1]

    # generate AUC as the classification score
    score_train = roc_auc_score(Y[train_index],prob_train)
    score_test = roc_auc_score(Y[test_index],prob_test)

    return score_train, score_test


result = e.classification(win_size='10ms',fold=15,
                          reshape_X_method=reshape_X,
                          run_model=run_model)
result.correct(method='cluster').plot()
```

Supplementary Result 1.
Name: Topography

**Samples in Data:
time   0   100   200   300   400 \
channel condition_group
AF3   0 S vs U   -0.161358 -0.083674 -0.914262 -1.752146 -1.136859
AF4   0 S vs U   -0.129856 -0.071146 -0.857713 -1.592307 -0.981774
AF7   0 S vs U   -0.198511 -0.038452 -0.696764 -1.502560 -0.602885
AF8   0 S vs U   -0.226421 -0.283624 -0.853643 -1.622599 -0.647796
AFz   0 S vs U   -0.133386 -0.024470 -0.847504 -1.709511 -1.275690

time   500   600
channel condition_group
AF3   0 S vs U   -0.325774 0.549780
AF4   0 S vs U   -0.011146 0.921798
AF7   0 S vs U   0.403305 1.168123
AF8   0 S vs U   0.546619 1.276115
AFz   0 S vs U   -0.526475 0.507121

**Samples in Annotation:
time   0   100   200   300   400 \
channel condition_group
AF3   0 S vs U   0.202987 0.546290 0.000096 6.792738e-06 0.003323

AF4	0 S vs U	0.301619	0.590314	0.000027	2.272653e-06	0.010381
AF7	0 S vs U	0.246107	0.884012	0.030994	1.328164e-03	0.196879
AF8	0 S vs U	0.109700	0.095464	0.000315	1.312931e-05	0.099305
AFz	0 S vs U	0.269374	0.820518	0.000017	3.118241e-07	0.000867

time		500	600
channel	condition_group		
AF3	0 S vs U	0.386503	0.112884
AF4	0 S vs U	0.980080	0.072064
AF7	0 S vs U	0.445874	0.020448
AF8	0 S vs U	0.353624	0.061543
AFz	0 S vs U	0.180809	0.200313

Supplementary Result 2.
Name: significant_channels_count

**Samples in Data:

time	2	7	12	17	22	27	32	37	42	47	...	552 \
condition_group											...	
0 S vs U	1	0	0	1	2	2	1	1	0	2	...	20
0 S vs F	1	1	4	6	6	4	2	1	1	0	...	36
0 F vs U	2	4	7	12	10	7	7	3	2	0	...	21

time	557	562	567	572	577	582	587	592	597
condition_group									
0 S vs U	20	25	27	27	34	35	35	34	35
0 S vs F	38	38	39	37	37	37	38	39	40
0 F vs U	23	25	24	23	25	26	28	29	28

[3 rows x 120 columns]

Supplementary Result 3.
Name: GFP

**Samples in Data:

time			0	1	2	3 \
subject	condition_group	channel_group				
1	0 Scrambled	0 All	0.377265	0.380017	0.384257	0.389661
10	0 Scrambled	0 All	0.557882	0.555313	0.553497	0.552378
11	0 Scrambled	0 All	0.515722	0.514311	0.513238	0.512541
12	0 Scrambled	0 All	0.957178	0.964533	0.971517	0.977753
13	0 Scrambled	0 All	0.508950	0.513307	0.519212	0.526837

time			4	5	6	7 \
subject	condition_group	channel_group				
1	0 Scrambled	0 All	0.396364	0.403958	0.412531	0.421655

10   0 Scrambled   0 All   0.552115 0.552640 0.554080 0.556310
11   0 Scrambled   0 All   0.512792 0.513938 0.516386 0.519909
12   0 Scrambled   0 All   0.982923 0.986924 0.989655 0.991306
13   0 Scrambled   0 All   0.536054 0.546944 0.559344 0.573259

time   8   9   ...   591 \
subject condition_group channel_group   ...
1   0 Scrambled   0 All   0.431422 0.441451 ...   2.826535
10   0 Scrambled   0 All   0.559373 0.563067 ...   3.074117
11   0 Scrambled   0 All   0.524721 0.530433 ...   3.410740
12   0 Scrambled   0 All   0.991966 0.992014 ...   5.054030
13   0 Scrambled   0 All   0.588519 0.605061 ...   4.355536

time   592   593   594   595 \
subject condition_group channel_group
1   0 Scrambled   0 All   2.842860 2.858723 2.873448 2.887393
10   0 Scrambled   0 All   3.082331 3.092524 3.104206 3.117395
11   0 Scrambled   0 All   3.408594 3.407195 3.405903 3.405528
12   0 Scrambled   0 All   5.021280 4.992827 4.967359 4.945983
13   0 Scrambled   0 All   4.308180 4.262083 4.215651 4.170986

time   596   597   598   599 \
subject condition_group channel_group
1   0 Scrambled   0 All   2.900004 2.911627 2.921911 2.931053
10   0 Scrambled   0 All   3.131661 3.146771 3.162507 3.178125
11   0 Scrambled   0 All   3.405432 3.406359 3.407715 3.410109
12   0 Scrambled   0 All   4.927431 4.913008 4.901544 4.894480
13   0 Scrambled   0 All   4.126118 4.083614 4.040732 4.001198

time   600
subject condition_group channel_group
1   0 Scrambled   0 All   2.939228
10   0 Scrambled   0 All   3.194377
11   0 Scrambled   0 All   3.413193
12   0 Scrambled   0 All   4.890638
13   0 Scrambled   0 All   3.959817

[5 rows x 601 columns]

**Samples in Annotation:
time   10   30   50   70   90   \
condition_group
0 Scrambled,0 Famous 0.329679 0.890589 0.331695 0.069825 0.074358

time   110   130   150   170   190 \
condition_group

```
0 Scrambled,0 Famous  0.177981  0.195641  0.002531  0.00984  0.000013

time                    ...         410      430       450       470  \
condition_group         ...
0 Scrambled,0 Famous    ...     0.020143  0.03582  0.028712  0.015977

time                      490       510       530       550      570  \
condition_group
0 Scrambled,0 Famous  0.001935  0.000462  0.000216  0.000077  0.00002

time                      590
condition_group
0 Scrambled,0 Famous  0.000033

[1 rows x 30 columns]
```

Supplementary Result 4.
Name: TANOVA

```
**Samples in Data:
time                  2         7         12        17        22        27  \
condition_group
0 S vs U        0.556444  0.745255  0.664336  0.644356  0.813187  0.816184
0 S vs F        0.617383  0.344655  0.222777  0.257742  0.453546  0.644356
0 F vs U        0.784216  0.547453  0.369630  0.256743  0.239760  0.295704

time                  32        37        42        47     ...         552  \
condition_group                                          ...
0 S vs U        0.634366  0.528472  0.508492  0.434565    ...     0.000999
0 S vs F        0.723277  0.729271  0.754246  0.850150    ...     0.000999
0 F vs U        0.356643  0.315684  0.264735  0.273726    ...     0.090909

time                 557       562       567       572       577       582  \
condition_group
0 S vs U        0.000999  0.000999  0.000999  0.000999  0.000999  0.000999
0 S vs F        0.000999  0.000999  0.000999  0.000999  0.000999  0.000999
0 F vs U        0.079920  0.047952  0.018981  0.005994  0.001998  0.001998

time                 587       592       597
condition_group
0 S vs U        0.000999  0.000999  0.000999
0 S vs F        0.000999  0.000999  0.000999
0 F vs U        0.001998  0.003996  0.009990

[3 rows x 120 columns]
```

**Samples in Annotation:
time 2 7 12 17 22 \
condition_group subject
0 S vs U 0 0.133383 0.100340 0.113674 0.110730 0.083330
0 S vs F 0 0.111794 0.147138 0.163269 0.138288 0.097395
0 F vs U 0 0.098390 0.121824 0.143361 0.156552 0.154418

time 27 32 37 42 47 \
condition_group subject
0 S vs U 0 0.078676 0.101468 0.114250 0.112197 0.114189
0 S vs F 0 0.070931 0.063608 0.065373 0.063950 0.054421
0 F vs U 0 0.135813 0.122365 0.130785 0.143962 0.135744

time ... 552 557 562 567 \
condition_group subject ...
0 S vs U 0 ... 0.013855 0.013904 0.014847 0.016371
0 S vs F 0 ... 0.022856 0.021666 0.022175 0.023972
0 F vs U 0 ... 0.002830 0.002985 0.003495 0.004459

time 572 577 582 587 592 \
condition_group subject
0 S vs U 0 0.017761 0.018312 0.017975 0.017381 0.017173
0 S vs F 0 0.026226 0.027683 0.027579 0.026578 0.026077
0 F vs U 0 0.005764 0.006984 0.007656 0.007570 0.006839

time 597
condition_group subject
0 S vs U 0 0.017697
0 S vs F 0 0.026799
0 F vs U 0 0.005814

[3 rows x 120 columns]

Supplementary Result 5.
Name: Pattern classification

**Samples in Data:
time 2 7 12 17 22 27 32 37 \
condition_group
0 S vs F 0.256 0.641 0.718 0.952 0.898 0.852 0.885 0.728
0 S vs U 0.232 0.343 0.787 0.929 0.904 0.867 0.540 0.497
0 U vs F 0.037 0.192 0.492 0.668 0.615 0.732 0.566 0.636

time 42 47 ... 552 557 562 567 572 577 582 \
condition_group ...
0 S vs F 0.857 0.951 ... 0.000 0.0 0.0 0.000 0.000 0.0 0.000

```
0 S vs U        0.263  0.609 ...   0.000  0.0  0.0  0.000  0.000  0.0  0.000
0 U vs F        0.306  0.401 ...   0.005  0.0  0.0  0.002  0.001  0.0  0.002

time            587  592  597
condition_group
0 S vs F        0.0  0.0  0.0
0 S vs U        0.0  0.0  0.0
0 U vs F        0.0  0.0  0.0

[3 rows x 120 columns]

**Samples in Annotation:
time                         2         7        12        17        22   \
condition_group subject
0 S vs F        1        0.555130  0.529193  0.497357  0.495715  0.493275
                10       0.503662  0.496073  0.499009  0.476619  0.484096
                11       0.488151  0.515271  0.504270  0.475102  0.458023
                12       0.447826  0.440999  0.424995  0.399981  0.399796
                13       0.463077  0.453845  0.486155  0.480674  0.463548

time                        27        32        37        42        47   \
condition_group subject
0 S vs F        1        0.513620  0.492651  0.508405  0.509078  0.515480
                10       0.474934  0.482625  0.484719  0.498233  0.514622
                11       0.441614  0.455741  0.457926  0.479990  0.481456
                12       0.426179  0.463571  0.442368  0.475930  0.527808
                13       0.488689  0.509775  0.499828  0.486003  0.486661

time                       ...       552       557       562       567  \
condition_group subject    ...
0 S vs F        1          ...   0.709275  0.688575  0.701784  0.705450
                10         ...   0.646246  0.633509  0.627727  0.649949
                11         ...   0.690209  0.685347  0.700046  0.704479
                12         ...   0.712914  0.683256  0.666494  0.681073
                13         ...   0.865731  0.857273  0.860178  0.868929

time                        572       577       582       587       592  \
condition_group subject
0 S vs F        1        0.703124  0.717007  0.710632  0.708465  0.709904
                10       0.650215  0.653917  0.663795  0.664939  0.640950
                11       0.712028  0.694857  0.689622  0.694704  0.666910
                12       0.678316  0.687863  0.690749  0.717743  0.693562
                13       0.869681  0.862189  0.852487  0.854441  0.863375

time                        597
condition_group subject
```

0 S vs F	1	0.698791
	10	0.625715
	11	0.661164
	12	0.700814
	13	0.857351

[5 rows x 120 columns]
